# The malaria parasite *Plasmodium falciparum* in red blood cells selectively takes up serum proteins that affect host pathogenicity

**DOI:** 10.1186/s12936-020-03229-1

**Published:** 2020-04-15

**Authors:** Takahiro Tougan, Jyotheeswara R. Edula, Masayuki Morita, Eizo Takashima, Hajime Honma, Takafumi Tsuboi, Toshihiro Horii

**Affiliations:** 1 Research Centre for Infectious Disease Control, Research Institute for Microbial Diseases, Osaka University, 3-1 Yamadaoka, Suita, Osaka 565-0871 Japan; 2 Department of Molecular Protozoology, Research Institute for Microbial Diseases, Osaka University, 3-1 Yamadaoka, Suita, Osaka 565-0871 Japan; 3 Division of Malaria Research, Proteo-Science Centre, Ehime University, 3 Bunkyo-cho, Matsuyama, Ehime 790-8577 Japan; 4 Department of International Affairs and Tropical Medicine, Tokyo Women’s Medical University, 8-1 Kawada-cho, Shinjuku-ku, Tokyo, 162-8666 Japan; 5 Department of Malaria Vaccine Development, Research Institute for Microbial Diseases, Osaka University, 3-1 Yamadaoka, Suita, Osaka 565-0871 Japan; 6 Present Address: Cell and Developmental Biology Section, Division of Biological Sciences, University of California, San Diego, 9500 Gilman Dr, La Jolla, CA 92093 USA

**Keywords:** *Plasmodium falciparum*, Serum protein uptake, Calcium ion, Thrombin, Blood coagulation

## Abstract

**Background:**

The malaria parasite *Plasmodium falciparum* is a protozoan that develops in red blood cells (RBCs) and requires various host factors. For its development in RBCs, nutrients not only from the RBC cytosol but also from the extracellular milieu must be acquired. Although the utilization of host nutrients by *P. falciparum* has been extensively analysed, only a few studies have reported its utilization of host serum proteins. Hence, the aim of the current study was to comprehensively identify host serum proteins taken up by *P. falciparum* parasites and to elucidate their role in pathogenesis.

**Methods:**

*Plasmodium falciparum* was cultured with human serum in vitro. Uptake of serum proteins by parasites was comprehensively determined via shotgun liquid chromatography–mass spectrometry/mass spectrometry and western blotting. The calcium ion concentration in serum was also evaluated, and coagulation activity of the parasite lysate was assessed.

**Results:**

Three proteins, vitamin K-dependent protein S, prothrombin, and vitronectin, were selectively internalized under sufficient Ca^2+^ levels in the culture medium. The uptake of these proteins was initiated before DNA replication, and increased during the trophozoite and schizont stages, irrespective of the assembly/disassembly of actin filaments. Coagulation assay revealed that prothrombin was activated and thereby induced blood coagulation.

**Conclusions:**

Serum proteins were taken up by parasites under culture conditions with sufficient Ca^2+^ levels. This uptake phenomenon was associated with their pathogenicity.

## Background

The malaria parasite *Plasmodium falciparum* is a protozoan that reproduces in red blood cells (RBCs) and requires various host factors for its development and survival. For instance, protozoan parasites, including *Plasmodium* spp., rely on salvaging purines from the host as they are unable to synthesize purine rings de novo [1]. Furthermore, the parasites acquire amino acids as well as iron ions from the haemoglobin of their host cells. Interestingly, haemoglobin does not contain isoleucine and is low in several amino acids, such as methionine, causing these amino acids to be imported from the extracellular milieu [2]. Although the uptake mechanism of nutrients from the extracellular milieu has been intensively analysed [3], few studies have reported the uptake of host serum proteins. For example, ovalbumin, *P. falciparum* histidine-rich protein 2, human serum albumin (HSA), β-galactosidase, β-amylase, and horseradish peroxidase (HRP) are taken up and digested by the parasite after addition to the parasite culture medium [4, 5]. Kininogen is also taken up and modified to form bradykinin as well as other kinins, via intracellular proteolysis, which then elicits a calcium response in human umbilical vein endothelial cells in vitro [6]. Plasminogen is taken up and hydrolysed, facilitating the production of active angiostatin-like fragments that function to modulate host physiology during infection [7]. Furthermore, Tougan et al. [8] demonstrated that vitronectin is taken up by parasite-infected RBCs (iRBCs) where it binds directly to the P47 domain of serine repeat antigen 5 (SERA5), thereby camouflaging the parasite and enabling its evasion of the host immune system.

Ca^2+^ is essential for parasite development during the erythrocytic stage [9]. Plasma Ca^2+^, specifically, contributes to merozoite invasion of RBCs, as well as parasite development in RBCs [10–12]. Cytoplasmic Ca^2+^ concentration has been shown to slowly increase during parasite development, activating both host and parasite proteases during the schizont stage, and inducing merozoite egress from iRBCs [13–15]. Furthermore, plasma Ca^2+^ is required for host blood coagulation [16]. Activation of blood coagulation is frequently observed in patients with malaria, which subsequently induces inflammation and severe malaria-associated symptoms. In fact, the degree of coagulation activation is proportional to the severity of disease-related symptoms such as fever and disseminated intravascular coagulation (DIC) [17, 18]. Clinically apparent DIC is associated with severe outcomes and high mortality rates. During severely complicated malarial infection, the intrinsic coagulation pathway is activated by thrombin generation, which is pivotal for activation of the coagulation cascade [19]. Activated thrombin cleaves the major parasite adhesive protein on the surface of iRBCs. Consequently, iRBC adhesion dramatically decreases, and adherent iRBCs detach [20].

In the present study, serum proteins taken up by *P. falciparum* were comprehensively identified. The associated mechanisms of serum proteins and their pathogenicity were also analysed. These analyses revealed that the parasites selectively take up serum proteins that are associated with malaria pathogenicity.

## Methods

### Reagents and antibodies

CaCl_2_ was prepared as a 1 M stock solution in saline (0.9% w/v sodium chloride; Otsuka Pharmaceutical, Tokushima, Japan). Chelators including, ethylenediaminetetraacetic acid (EDTA), ethylene glycol-bis(β-aminoethyl ether)-*N*,*N*,*N*ʹ,*N*ʹ-tetraacetic acid (EGTA), and tri-sodium citrate dihydrate (sodium citrate) were purchased from Nacalai Tesque (Kyoto, Japan) and prepared as a 100 mM stock solution in saline. Heparin sodium (1000 IU/mL) was obtained from Mochida Pharmaceutical (Tokyo, Japan). Jasplakinolide (Jas; Abcam, Cambridge, UK), cytochalasin D (CytD; Wako, Osaka, Japan), and mycalolide B (MycB; Wako) were prepared as 10 mM stock solutions in dimethyl sulfoxide (DMSO; Nacalai Tesque). Phenylmethylsulfonyl fluoride (PMSF; Nacalai Tesque) and dabigatran etexilate (DE; Selleck Chemicals LLC, Houston, TX, USA) were prepared as 1 mM stock solutions in DMSO.

Mouse anti-SE36 and rabbit anti-EXP2/3C sera were prepared accordingly to previously described protocols [21, 22]. Anti-protein S polyclonal antibody (pAb) (16910-1-AP), anti-prothrombin pAb (24295-1-AP), anti-vitronectin pAb (15833-1-AP), anti-alpha-2-HS-glycoprotein pAb (16571-1-AP), anti-alpha-2-macroglobulin pAb (13545-1-AP), anti-band 3 pAb (18566-1-AP), anti-fibrinogen beta chain pAb (16747-1-AP), and rabbit control IgG (30000-0-AP) were obtained from Proteintech (Rosemont, IL, USA). Anti-C4b-binding protein alpha chain monoclonal antibody (mAb) (ab182140), anti-C5 pAb (ab46153), anti-apolipoprotein A-I pAb (ab7613), anti-apolipoprotein B-100 pAb (ab7616), anti-thrombin mAb (ab92621), and anti-Factor Xa pAb (ab111171) were obtained from Abcam. Anti-HSA pAb (A80-129P) was purchased from Bethyl Laboratories (Montgomery, TX, USA). Anti-human IgG heavy chain pAb (709-035-149) was procured from Jackson ImmunoResearch Laboratories (West Grove, PA, USA). Anti-β-Actin pAb (PM053-7) was purchased from MBL (Nagoya, Japan). Secondary antibodies, namely HRP- and fluorescence-conjugated secondary antibodies [anti-mouse IgG-HRP (115-035-166), anti-rabbit IgG-HRP (711-035-152), anti-goat IgG-HRP (805-035-180), and anti-rabbit IgG-Alexa Fluor 594 (711-585-152)] were purchased from Jackson ImmunoResearch Laboratories.

### Serum preparation

The following methods were employed for serum preparation (Additional file 1: Fig. S1). (1) Plasma-derived serum (PDS): plasma was collected from blood samples using a blood bag containing citrate-phosphate-dextrose solution (Terumo, Tokyo, Japan). Plasma and buffy coat (approximately 400 mL) were coagulated by the addition of 1 M CaCl_2_, followed by incubation at 37 °C for 4 h and subsequently at 4 °C overnight. (2) Naturally coagulated serum (NCS): blood samples were allowed to coagulate naturally at 37 °C for 1 h. After coagulation, serum was collected by centrifugation at 10,400×*g* for 20 min at 4 °C, followed by filtration through a 0.45-µm filter. Sera were then incubated at 56 °C for 30 min for complement inactivation. The concentration of Ca^2+^ in serum was measured using the Metallo assay kit LS (CPZIII; Metallogenics, Chiba, Japan), according to the manufacturer’s instructions.

### Parasite culture

*Plasmodium falciparum* strain 3D7 was cultured in RPMI 1640 medium supplemented with l-glutamine (0.5 g/L), HEPES (5.95 g/L), NaHCO_3_ (2 g/L), hypoxanthine (50 mg/L), gentamicin (10 mg/L), human serum (10%), and RBCs (haematocrit, 3%) in an atmosphere of 5% CO_2_, 5% O_2_, and 90% N_2_ at 37 °C, as described previously [23].

### Uptake assay

Ring stage-synchronized iRBCs were collected using the sorbitol synchronization technique [24]. The RBCs were collected using centrifugation at 800×*g* for 5 min. The supernatant was discarded, and the cells were suspended in 5× volume of 5% d-sorbitol. The mixture was incubated for 10 min at room temperature (18–25 °C). The cells were washed twice with RPMI 1640 to remove the sorbitol and adjusted to a parasitaemia level of approximately 1.5% at 3% haematocrit. The collected iRBCs were continuously cultured for approximately 18 h. To prepare the parasite lysate, the following two methods were used. (1) Percoll method: the cultured iRBCs were collected by Percoll density gradient centrifugation (Percoll, 58.5%), as described previously by Tosta et al. [25]. (2) Precipitation method: RBCs were collected and washed with saline by centrifugation at 800×*g* for 5 min at 4 °C. The resulting precipitate was lysed using 0.075% saponin in saline, and the lysate (designated as “parasite”) was collected and washed using saline by centrifugation at 10,400×*g* for 10 min at 4 °C. For the Percoll method, the supernatant containing RBC content (designated as “RBC”) was also collected and was used for western blotting.

### Shotgun liquid chromatography–mass spectrometry/mass spectrometry (LC–MS/MS) analysis

The samples prepared by the Percoll method were subjected to SDS-PAGE, excised from the gel, and reduced with 10 mM dithiothreitol, followed by alkylation with 55 mM iodoacetamide and in-gel digestion with trypsin. The prepared samples were analysed using nanocapillary reversed-phase LC–MS/MS with a Magic C18 reverse phase column (0.1  ×  150 mm) on a nano LC system (Thermo Fisher Scientific, Waltham, MA, USA) coupled to a quadrupole time-of-flight mass spectrometer (QTOF Ultima; Waters, Milford, MA, USA). Direct injection data-dependent acquisition was performed using one MS channel for every three MS/MS channels and dynamic exclusion for selected ions. The ion mode was set to positive. The dynamic exclusion was set to 30 s with 10 ppm tolerance. Proteins were identified by searching in the Swiss-Prot Human database using the Mascot Server (Matrix Science, Boston, MA, USA). A Mascot peptide significance threshold of p < 0.05 was used for post search filtering. MS error window was 10 ppm and MS/MS was 0.8 Da. To determine the uptake ratio of individual serum proteins, non-serum proteins (such as keratin, RBC component, and immunoglobulin light chains) were excluded from the analysis. The total spectral count (TSC) of each serum protein within the intact parasite lysate was individually compared with the TSC of a corresponding serum protein. The uptake ratio was calculated as follows: uptake ratio = TSC of isolated parasite lysate protein/TSC of serum protein.

### Protease protection assay

To study the uptake of serum proteins into parasitophorous vacuoles (PV), a protease protection assay was performed as previously described [26, 27]. In brief, the cultured iRBCs were collected by the Percoll method, and the RBC membranes were lysed with 300 U streptolysin O (Sigma-Aldrich, St. Louis, MO, USA) for 10 min at room temperature (18–25 °C). The treated samples were centrifuged at 1000×*g* for 4 min at room temperature (18–25 °C) and the supernatant (designated as “RBC”) and pellet (designated as “PV”) were separated. This step was repeated twice and the pellet was suspended in phosphate-buffered saline (PBS) at a volume equal to that of the supernatant. The supernatant and pellet were treated either with 0.1 mg/mL proteinase K (Sigma-Aldrich) or PBS as a control for 30 min on ice followed by treatment with 2 mM PMSF and 1× protease inhibitor cocktail (Nacalai Tesque) for 3 min at room temperature (18–25 °C) to prevent further protease activity and were then used for western blotting.

### Western blotting

The sera and lysate samples, purified by the Percoll or precipitation method, were suspended in sample buffer [NuPAGE LDS Sample Buffer (4×)] supplemented with a reducing reagent [NuPAGE Sample Reducing Agent (10×)] and resolved on a NuPAGE 4%–12% Bis-Tris gel using 1× NuPAGE MES SDS Running Buffer (Thermo Fisher Scientific). The separated proteins were transferred onto polyvinylidene fluoride membranes using iBlot (Thermo Fisher Scientific). The membranes were blocked with blocking buffer [PBS containing 0.05% (v/v) Tween 20 (PBS-T) with 5% (w/v) skim milk] for 30 min, and then incubated in blocking buffer containing primary antibodies or antiserum (all antibodies and antiserum were diluted 1:2000; save for the anti-prothrombin pAb and the anti-human IgG pAb-HRP, which were diluted 1:10,000) for 1 h. After washing with PBS-T, the membranes were incubated in blocking buffer containing the respective secondary antibody (1:10,000; anti-mouse IgG-HRP, anti-rabbit IgG-HRP, or anti-goat IgG-HRP) for 30 min. The membranes were washed with PBS-T, soaked in TMB Microwell Peroxidase Substrate System (KPL, Gaithersburg, MD, USA), and analysed using a LAS 4000 (GE Healthcare, Little Chalfont, UK). Densitometry of the observed bands was performed using ImageJ software, version 1.8.0 (NIH, Bethesda, MD, USA).

### Imaging

**Fig. 1:**
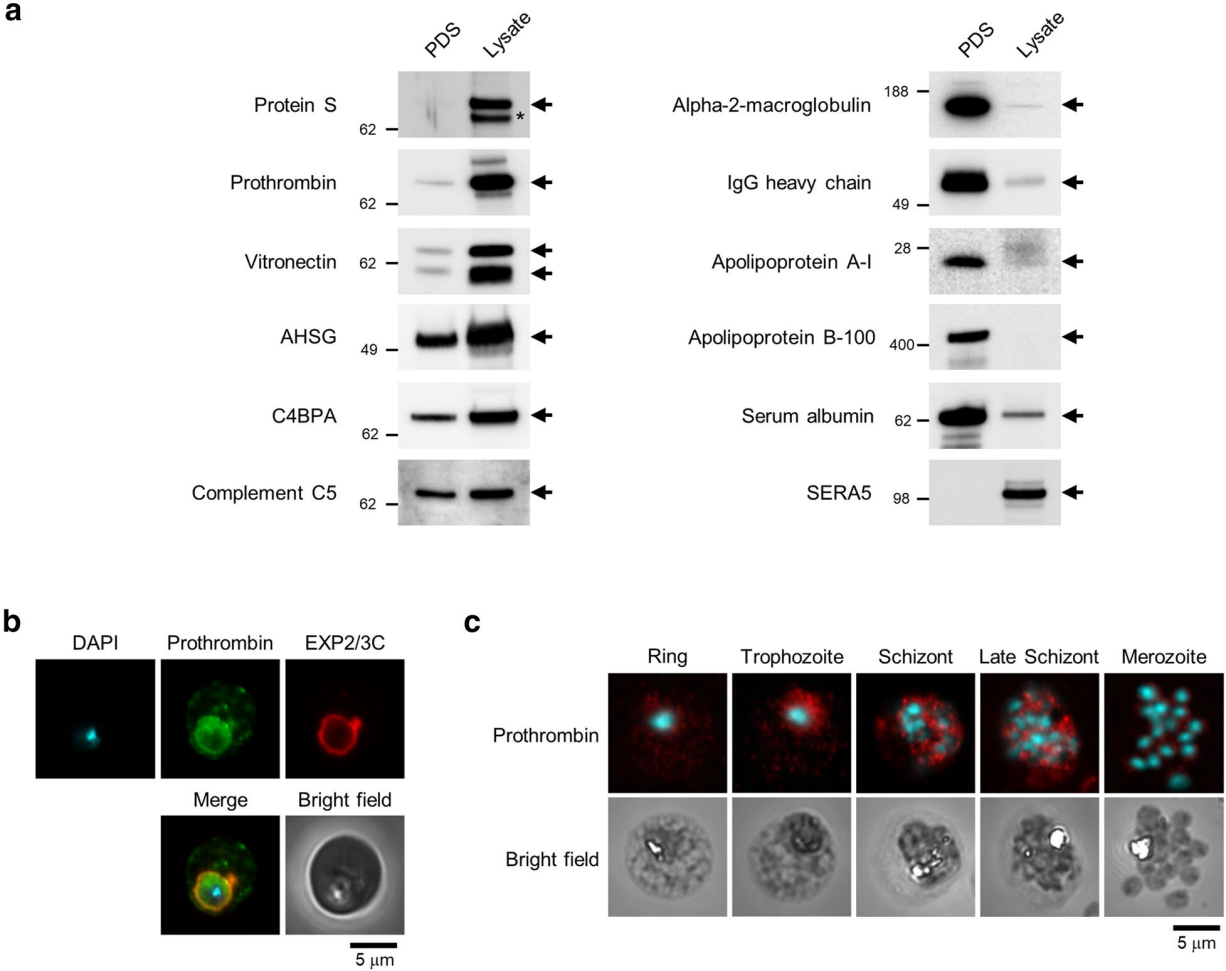
Serum proteins were selectively taken up by *Plasmodium falciparum*. **a** Western blotting analysis of serum proteins. “Serum” and “Lysate” indicate plasma-derived serum (PDS) and isolated parasite lysate prepared from infected red blood cells (iRBCs) cultured in PDS-containing medium. Samples were prepared using the Percoll method. SERA5 was used as an internal control. *Protein S* vitamin K-dependent protein S, *AHSG* alpha-2-HS-glycoprotein, *C4BPA* C4b-binding protein alpha chain, *SERA5* serine repeat antigen 5. The asterisk indicates a non-specific band. **b** Confocal microscopy images showing localization of prothrombin (green), EXP2/3C (red), and DNA (cyan). Samples were fixed using the paraformaldehyde/glutaraldehyde fixation method. Scale bar represents 5 µm. **c** Confocal microscopy images showing the localization of prothrombin (red) and DNA (cyan). Samples were fixed using the methanol fixation method. The scale bar represents 5 µm. All samples were prepared from 3D7 strain

For confocal microscopy, the following two methods were adopted for sample preparation. (1) Methanol fixation: thin iRBC smears on glass slides were fixed using 100% methanol for 10 min, permeabilized using PBS containing 0.1% Triton X-100 for 5 min and washed with PBS. Samples were blocked using a blocking buffer (1% BSA in PBS) for 30 min, followed by incubation for 1.5 h in blocking buffer containing the respective primary antibodies (1:200; anti-protein S, prothrombin, or vitronectin pAb). After washing with PBS, samples were incubated in blocking buffer containing secondary antibodies (1:200; anti-rabbit IgG-Alexa Fluor 594) for 1.5 h. (2) Paraformaldehyde/glutaraldehyde fixation: iRBCs were pelleted, washed with PBS, and fixed in 4% paraformaldehyde and 0.0075% glutaraldehyde, as described by Tonkin et al. [28]. They were then treated with blocking buffer (3% BSA in PBS) for 30 min and subsequently incubated for 1 h in blocking buffer containing the respective primary antibodies or antiserum (1:200, anti-prothrombin pAb; 1:1000, anti-EXP2/3C mouse serum). After washing with PBS, the slides were incubated in blocking solution containing the respective secondary antibodies (1:1000; anti-rabbit IgG-Alexa Fluor 488 or anti-mouse IgG-Alexa Fluor 568) for 1 h. The iRBCs were then transferred onto 1% polyethylenimine (PEI)-coated cover glass for 30 min. The samples were mounted using Vectashield with DAPI (Vector Labs, Burlingame, CA, USA). All samples were fixed using methanol fixation, save for those shown in Fig. 1b. Images were captured using an Olympus FV10i (Olympus, Tokyo, Japan).

### Determination of parasite state

Light microscopy: a standard thin blood smear was fixed with 100% methanol for 10 min and stained with 10% Giemsa working solution, pH 7.2 (Merck KGaA, Darmstadt, Germany), for 13 min. Slides were observed under 1000× magnification using a model BX50 light microscope (Olympus, Tokyo, Japan). An automated haematology analyser XN-30 equipped with an algorithm for cultured *P. falciparum* parasites (prototype: software version 01-03, build 16) was used with dedicated reagents (CELLPACK DCL, SULFOLYSER, Lysercell M, and Fluorocell M) (Sysmex, Kobe, Japan) [29]. Approximately 100 µL of culture suspension was aliquoted into a standard 1.5 mL microtube (Eppendorf Tubes 3810X; Eppendorf, Hamburg, Germany), and loaded into the XN-30 analyser as described in the manufacturer’s manual (Sysmex). Parasitaemia (total, MI-RBC%; ring-form, RNG-RBC%; trophozoite, TRPZ-RBC%; and schizont, SCHZ-RBC%) was automatically reported [29]. However, as the XN-30 analyser detects DNA content, parasitaemia was overestimated due to the presence of dead parasites. Therefore, the M scattergram and parasitaemia level of parasites treated with antimalarial compounds must be compared with those of the negative control (DMSO) [30].

### Coagulation assay

Fifty millilitres of NCS was incubated in saline supplemented with 10% culture medium, culture supernatant, or parasite lysate in 100 µL reaction buffer (20 mM Tris–HCl, 100 mM NaCl, with or without 2.5 mM CaCl_2_, pH 8.0). To inhibit the protease activity of thrombin, 10 µM PMSF or 10 µM DE was added to the reaction mixture, which was then incubated for 2 h at 37 °C. The supernatants were collected by centrifugation at 12,000×*g* for 5 min at 4 °C and were used for western blotting.

## Results

### Serum proteins are selectively taken up into iRBCs by *P. falciparum* parasites

**Fig. 2:**
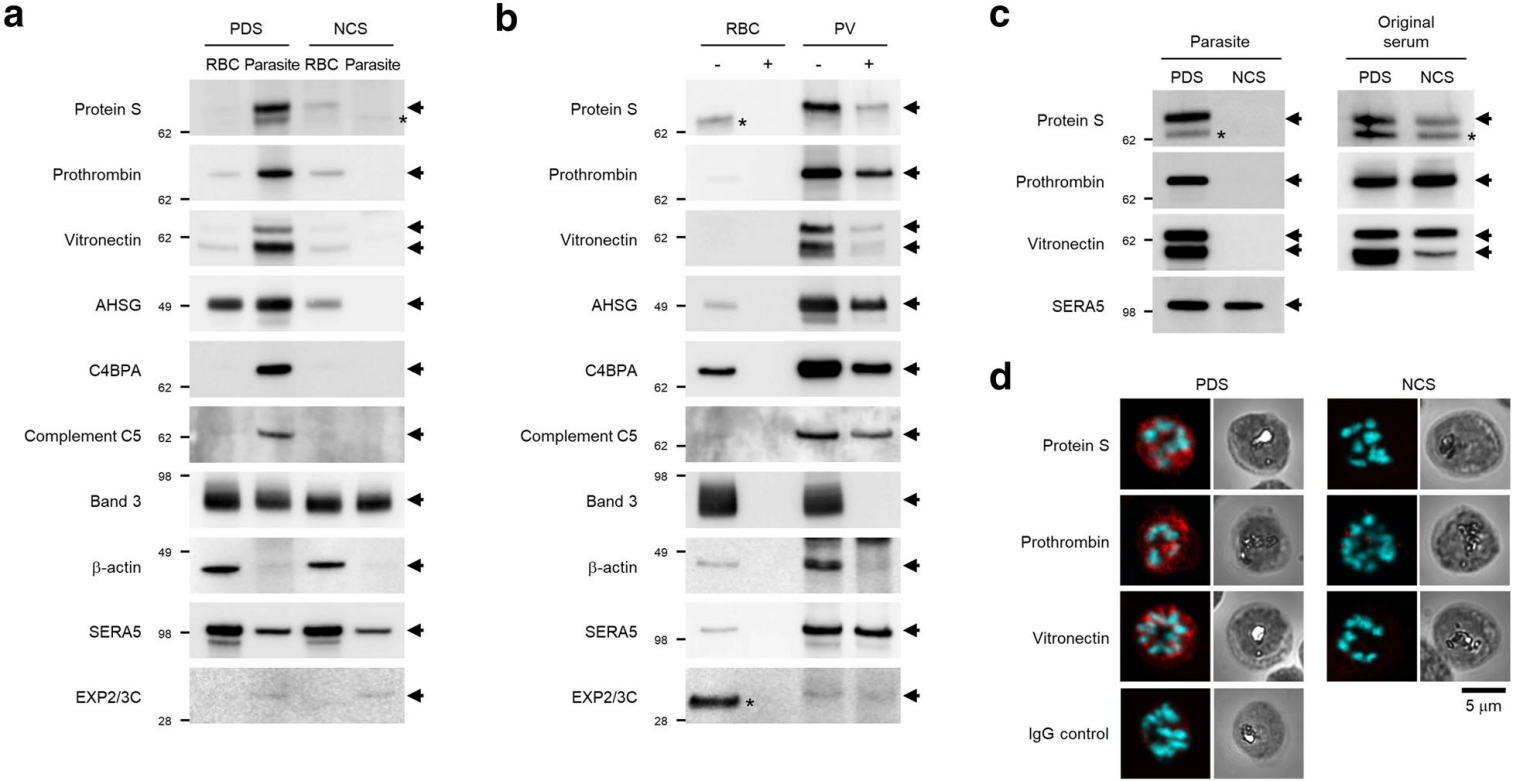
Uptake ability was dependent on the sample preparation method. **a**–**c** Western blotting analysis of human and parasite proteins. **a** Lysates prepared from iRBCs cultured in PDS- and naturally coagulated serum (NCS)-containing culture media. Samples were collected using the Percoll method and were treated with saponin. Band 3, β-actin, SERA5, and EXP2/3C were used as internal controls. **b** Protease protection assay in the RBC and PV lysates prepared from iRBCs cultured in PDS-containing culture medium. Samples were collected using the Percoll method and were treated with streptolysin O. “+” and “−” indicate treatment with or without proteinase K, respectively. Band 3, β-actin, SERA5, and EXP2/3C were used as internal controls. **c** (left panels) Serum proteins in the iRBC lysates cultured in PDS- and NCS-containing culture media. Samples were collected using the precipitation method and were treated with saponin. (right panels) Serum proteins in the original sera, PDS, and NCS. SERA5 was used as internal control. The asterisk indicates a non-specific band. **d** Confocal microscopy images showing the localization of protein S, prothrombin, vitronectin (red), and DNA (cyan) in iRBCs cultured in PDS- or NCS-containing medium. Samples were fixed using the methanol fixation method. As a control, rabbit control IgG was used instead of primary antibodies. The scale bar represents 5 µm. All samples were prepared from 3D7 strain

The total protein in plasma-derived serum (PDS), and within intact parasites in iRBCs cultured in PDS-containing culture medium was analysed by shotgun LC–MS/MS analysis. A total of 50 and 48 serum proteins were identified in the serum and isolated parasite lysate, respectively (Table 1, total spectral counts (TSCs) were 2.38–1850 and 0.86–129 in the serum and isolated parasite lysate, respectively). Comparative analysis revealed that vitamin K-dependent protein S (protein S) and prothrombin were taken up by intact parasites in a highly selective manner (Table 1; uptake ratio: protein S, 26.10; prothrombin, 10.36). The results of western blotting analysis were similar to those of the shotgun LC–MS/MS analysis (Fig. 1a). Although the uptake ratio of vitronectin was relatively low (3.99; Table 1) it was internalized in a strongly selective manner (Fig. 1a), as previously reported [8]. These findings suggest that serum proteins were selectively taken up, rather than simply diffusing into iRBCs. Further, confocal microscopy showed that prothrombin was localized in iRBCs, most notably within parasitophorous vacuoles (PVs), during the trophozoite stage (Fig. 1b). Specifically, prothrombin was detected in iRBCs during development (Figs. 1c and 2b, PDS panels). Uptake of protein S, prothrombin, and vitronectin was also observed in the blood samples prepared from mice infected with the rodent malaria parasite *Plasmodium yoelii* (Additional file 1: Fig. S2), suggesting that similar uptake mechanisms are present among *Plasmodium* spp.

**Table 1:** List of serum proteins internalized in malaria-infected red blood cells (iRBCs)

#	Identified proteins	Serum^a^	Lysate^b^	Uptake ratio^c^
1	Vitamin K-dependent protein S^d^	2.38	62.12	26.10
2	Prothrombin^d^	13.08	135.46	10.36
3	Vitronectin^d^	4.76	18.98	3.99
4	Alpha-2-HS-glycoprotein^d^	8.32	28.47	3.42
5	C4b-binding protein alpha chain^d^	28.54	50.04	1.75
6	Complement C5^d^	32.1	37.1	1.16
7	Hornerin	2.38	2.59	1.09
8	Apolipoprotein L1	3.57	3.45	0.97
9	Complement component C9	4.76	3.45	0.72
10	Complement C1q subcomponent subunit C	3.57	1.73	0.48
11	Antithrombin-III	35.67	15.53	0.44
12	Insulin-like growth factor-binding protein complex acid labile subunit	5.95	2.59	0.44
13	Clusterin	4.76	1.73	0.36
14	Protein AMBP	7.13	2.59	0.36
15	Heparin cofactor 2	19.03	6.9	0.36
16	Plasma protease C1 inhibitor	17.84	6.04	0.34
17	Fibronectin	73.72	22.43	0.30
18	Apolipoprotein(a)	5.95	1.73	0.29
19	Inter-alpha-trypsin inhibitor heavy chain H1	47.56	13.8	0.29
20	Plasminogen	28.54	7.77	0.27
21	Alpha-1-antitrypsin	93.94	23.3	0.25
22	Immunoglobulin lambda-like polypeptide 5	21.4	5.18	0.24
23	Complement factor B	32.1	7.77	0.24
24	Ig mu chain C region	36.86	8.63	0.23
25	Alpha-1-antichymotrypsin	28.54	6.04	0.21
26	Inter-alpha-trypsin inhibitor heavy chain H2	48.75	9.49	0.19
27	Inter-alpha-trypsin inhibitor heavy chain H4	35.67	6.9	0.19
28	Complement C3	330.56	52.63	0.16
29	Ceruloplasmin	49.94	7.77	0.16
30	Ig alpha-1 chain C region	59.45	7.77	0.13
31	Complement component C6	7.13	0.86	0.12
32	Alpha-2-macroglobulin^d^	222.36	26.75	0.12
33	Complement C4-B	136.74	16.39	0.12
34	Complement C4-A	137.93	16.39	0.12
35	Ig gamma-1 chain C region^d^	156.96	15.53	0.10
36	Histidine-rich glycoprotein	17.84	1.73	0.10
37	Apolipoprotein E	17.84	1.73	0.10
38	Apolipoprotein A–I^d^	54.7	5.18	0.09
39	Serum paraoxonase/arylesterase 1	19.03	1.73	0.09
40	Ig gamma-3 chain C region^d^	107.02	8.63	0.08
41	Ig gamma-2 chain C region^d^	114.15	8.63	0.08
42	Complement C1s subcomponent	11.89	0.86	0.07
43	Serum albumin^d^	1850.19	128.56	0.07
44	Apolipoprotein B-100^d^	363.85	15.53	0.04
45	Alpha-1B-glycoprotein	21.4	0.86	0.04
46	Gelsolin	22.59	0.86	0.04
47	Kininogen-1	30.92	0.86	0.03
48	Haptoglobin	60.64	0.86	0.01
49	Serotransferrin	259.22	0	0
50	Ig gamma-4 chain C region^d^	80.86	0	0

^a^Total spectral count (TSC) of serum protein

^b^TSC of isolated parasite lysate protein

^c^Uptake ratio = TSC of isolated parasite lysate protein/TSC of serum protein

^d^Uptake of protein was confirmed by western blotting in Fig. 1a

### Uptake ability is dependent on the serum preparation method

To characterize the ability of *P. falciparum* to take up proteins, the parasites were cultured in a medium containing two types of serum: PDS and naturally coagulated serum (NCS) (see serum preparation in “Methods” and Additional file 1: Fig. S1). Serum proteins were only detected in the parasite lysate isolated from iRBCs cultured in PDS-containing culture medium but not in the lysate isolated from iRBCs cultured in NCS-containing medium (Fig. 2a). Further, proteins identified in the lysate from protease-treated PV partially resisted digestion, suggesting that these proteins were taken up by parasites (Fig. 2b). The enhanced uptake in parasites cultured in the PDS-containing culture medium was confirmed via detection of protein S, prothrombin, and vitronectin within intact parasite lysates (Fig. 2c). The difference in uptake activity between parasites cultured in PDS- and NCS-containing culture media was also observed in the CDC1 and W2 strains (Additional file 1: Fig. S3). In addition, confocal microscopy confirmed that protein S, prothrombin, and vitronectin were localized in parasites within RBCs cultured in PDS-, not NCS-containing culture media, indicating that uptake ability is dependent on the serum preparation method.

### Plasma Ca^2+^ increased the uptake ability of parasites

**Fig. 3:**
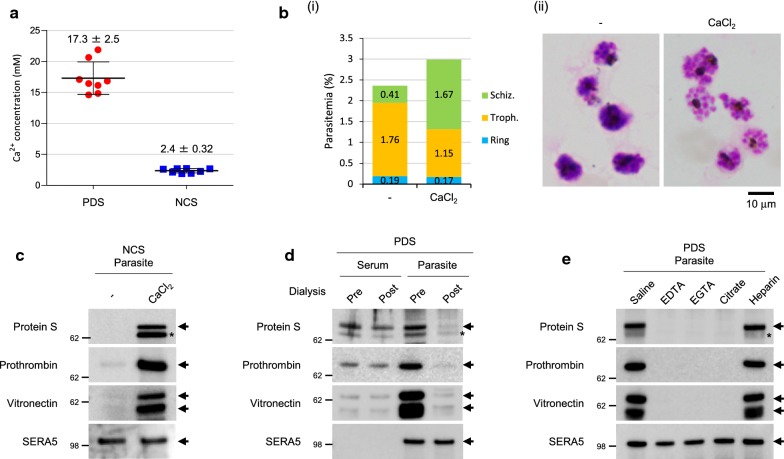
The serum proteins were taken up under sufficient Ca^2+^ levels in the culture medium. **a** Concentration of Ca^2+^ in PDS and NCS. Data represents the mean ± standard deviation (SD) for eight serum samples prepared using each method. **b** Comparison of parasitaemia and morphology of parasites cultured in NCS-containing medium with and without 2 mM CaCl_2_. (i) Parasitaemia. The ring stage-synchronized parasite culture (approximately 1% parasitaemia) was incubated with or without 2 mM CaCl_2_ for 48 h. (ii) Light microscopy images of parasites. The scale bar represents 10 µm. **c**–**e** Western blotting analysis of serum proteins. Samples were prepared using the precipitation method and were treated with saponin. **c** Uptake of serum proteins after the addition of 2 mM CaCl_2_ to NCS. **d** Uptake of serum proteins before (pre) and after (post) removal of Ca^2+^ from PDS by dialysis. **e** Uptake of serum proteins after removal of Ca^2+^ by the addition of 2.5 mM chelators (EDTA, EGTA, or sodium citrate). Approximately 5 IU/mL heparin, a non-chelating reagent, was added as a negative control. The asterisk indicates a non-specific band. All samples were prepared from 3D7 strain

The Ca^2+^ concentration in PDS and NCS was 17.3 ± 2.5 and 2.4 ± 0.32 mM, respectively (Fig. 3a); the Ca^2+^ concentration in normal serum is 2.2–2.6 mM [31]. The Ca^2+^ concentration in PDS was approximately eightfold higher than that in NCS, whereas that in NCS was within the normal range (Fig. 3a). This finding indicated that the Ca^2+^ concentration in the PDS-containing culture medium was similar to that in normal serum since the culture medium contained 10% human serum (see parasite culture in “Methods”). The importance of the Ca^2+^ concentration of the culture medium for protein uptake was confirmed by the following strategies: (1) addition of Ca^2+^ to NCS; and (2) removal of Ca^2+^ from PDS. The addition of CaCl_2_ to the NCS-containing culture medium was expected to slightly improve parasite growth (Fig. 3b); however, the SERA5 expression levels were similar in the media with and without CaCl_2_ (see SERA5 in Fig. 3c). Additionally, the uptake ability was notably increased by the addition of CaCl_2_ to NCS (Fig. 3c), and decreased by the removal of Ca^2+^ from PDS (Fig. 3d), and in PDS-containing culture medium treated with Ca^2+^ chelators, EDTA, EGTA, and sodium citrate, but not with heparin that is not a Ca^2+^ chelator (Fig. 3e). These results indicate that a sufficient concentration of Ca^2+^ is required for uptake.

### Uptake is initiated before DNA replication, irrespective of assembly/disassembly of actin filaments

**Fig. 4:**
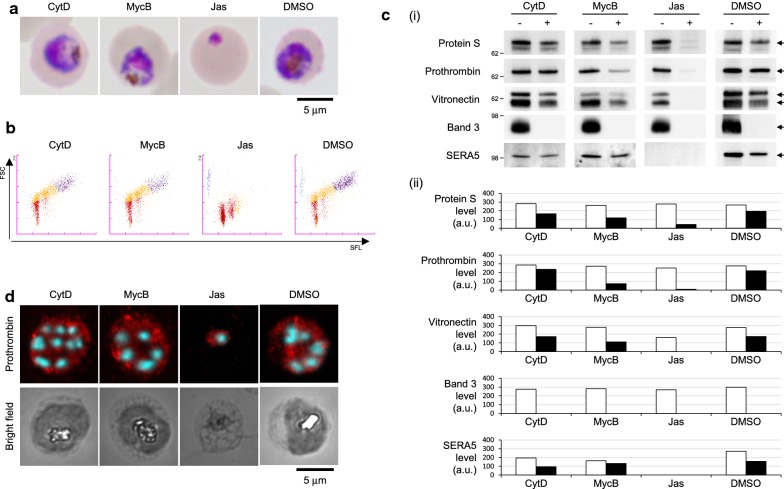
Assembly/disassembly of actin filaments was not required for the uptake of serum proteins. **a** Light microscopy images of parasites treated with actin-perturbing agents. The scale bar represents 5 µm. **b** M scattergrams showing the developmental stages of parasites after treatment. The horizontal and vertical axes represent DNA content and iRBC size, respectively. Colours indicate the following: red, ring-form; orange, trophozoite; purple, schizont; and blue, polychromatic red blood cell. These were assigned based on the default settings of the XN-30 analyser. **c** (i) Western blotting analysis of serum proteins. Samples were collected using the Percoll method and were treated with streptolysin O. “+” and “−” indicate treatment with or without proteinase K, respectively. (ii) Densitometry analysis of the observed bands in (i). Closed and open columns indicate treatment with or without proteinase K, respectively. *a.u.* arbitrary unit. Band 3 and SERA5 were used as internal controls. **d** Confocal microscopy images showing the localization of prothrombin (red) and DNA (cyan) after treatment. Samples were fixed using the methanol fixation method. Scale bar represents 5 µm. *CytD* cytochalasin D, *MycB* mycalolide B, *Jas* jasplakinolide. Each compound was added to the parasite culture at a final concentration of 50 µM. All experiments were performed in triplicate and representative data are shown

To further understand the uptake mechanism, three actin-perturbing agents, cytochalasin D (CytD), mycalolide B (MycB), and jasplakinolide (Jas) were added to the PDS-containing culture medium. CytD inhibits new assembly and MycB disassembles actin filaments, whereas Jas increases the assembly and stabilization of actin filament [32–34]. Parasite development was not noticeably affected by CytD and MycB, however, was arrested by Jas before DNA replication (Fig. 4a, b). Further, the uptake of serum proteins into the PV was slightly reduced by CytD and MycB treatment, suggesting that uptake occurs without the assembly/disassembly of actin filaments (Fig. 4c, d, CytD and MycB). Additionally, following Jas treatment serum proteins were detected at low levels in PV lysates (Fig. 4c, d, Jas) even though Jas arrested parasite development at the early stage (Fig. 4a, b, Jas and c, SERA5), suggesting that uptake is induced before DNA replication, as also shown in Fig. 1c.

### Blood coagulation is induced by thrombin activated by parasite protease

**Fig. 5:**
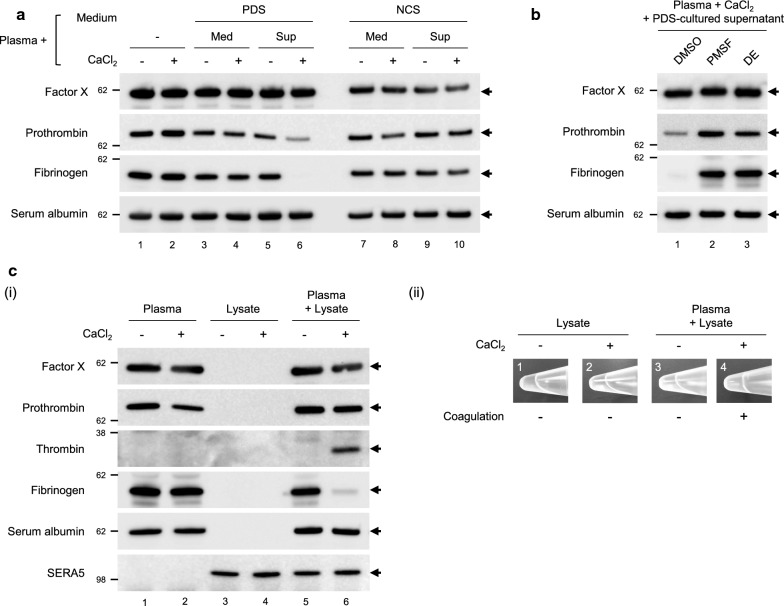
Coagulation occurred after parasite egress from iRBCs. **a**–**c** (i) Western blotting analysis of coagulation. **a** “Med” and “Sup” indicate PDS- or NCS-containing medium and PDS- or NCS-cultured supernatant, respectively. **b** After the addition of protease inhibitors (*PMSF* phenylmethylsulfonyl fluoride, *DE* dabigatran etexilate). **c** “Lysate” indicates the isolated parasite lysate prepared using the precipitation method. The isolated parasite lysate prepared from iRBCs cultured in NCS-containing medium was added to plasma. (i) The absence of fibrinogen indicated coagulation, save for in the isolated parasite lysate (lanes 3 and 4). (ii) Visual confirmation of coagulation. Tubes, shown in lanes 3, 4, 5, and 6 in (i), containing 100 µL of the reaction mixture were rotated at a 90° angle onto their sides after completion of the coagulation reaction. All experiments were performed in triplicate and representative data are shown

A coagulation assay was performed to evaluate the effect of serum proteins taken up in iRBCs, on the host. The addition of PDS-culture supernatant but not PDS-containing medium alone and NCS-culture supernatant induced coagulation in a CaCl_2_-dependent manner (Fig. 5a, lanes 6, 4, and 10; fibrinogen was not detected after coagulation). These results suggest that internalized prothrombin was activated and caused the conversion of fibrinogen to fibrin. To test this hypothesis, a coagulation assay with two protease inhibitors, PMSF (a common serine protease inhibitor) and DE (a thrombin-specific protease inhibitor) was performed. Coagulation was inhibited by both PMSF and DE, suggesting that coagulation was induced by protease activity of thrombin activated by isolated parasite lysate (Fig. 5b, lane 3). Finally, to determine whether parasite-derived proteases produce thrombin from prothrombin and induce coagulation, a coagulation assay using parasite lysate isolated from iRBCs cultured in NCS-containing medium was performed. The addition of isolated parasite lysate to plasma-generated thrombin cleaved fibrinogen in a Ca^2+^-dependent manner (Fig. 5c, lane 6 in (i) and image 4 in (ii)); however, coagulation was not induced in the plasma or isolated parasite lysate alone, even in the presence of Ca^2+^ (Fig. 5c, lane 2 and 4 in (i) and image 2 in (ii)). Furthermore, Factor X, a prothrombin activator, was not internalized (Fig. 5c (i)), and Factor Xa (cleaved and activated Factor X) was not detected (Additional file 1: Fig. S4, blot was overexposed to highlight the absence of Factor Xa). These findings suggested that the thrombin generated by an unidentified parasite protease induced coagulation in a Ca^2+^-dependent manner.

## Discussion

The current study identified 50 and 48 proteins in the serum and isolated parasite lysate, respectively (Table 1, TSCs were 1850–2.38 in serum and 129–0.86 in isolated parasite lysate). The dynamic ranges of TSC were approximately 800-fold and 150-fold in serum and isolated parasite lysate, respectively. This reduced dynamic range in the lysate suggests that serum proteins were selectively taken up in iRBCs. The uptake ratio of seven selectively internalized serum proteins ranged from 26.10 to 1.09; protein S, prothrombin, and vitronectin had particularly high uptakes ratios (26.10, 10.36, and 3.99, respectively; Fig. 1 and Table 1). Conversely, the uptake ratios of serum albumin, and kininogen, plasminogen, which were previously reported to be internalized in iRBCs [4, 6, 7], were lower (0.07, 0.03, and 0.27, respectively; Table 1), while transferrin [35] was not detected (Table 1). This discrepancy may be due to the fact that our proteomic analysis did not detect rapidly degraded proteins in the digestive vacuole as the parasites procure amino acids such as isoleucine from the extracellular milieu [2]; in this case, their uptake ratio would be underrepresented in this analysis.

The uptake of serum proteins was CaCl_2_ concentration-dependent (Fig. 3) as protein trafficking requires Ca^2+^, which is essential for vesicle formation and fusion, protein conformation, and cytoskeleton remodelling [36]. In addition, Ca^2+^ signalling pathways are critical throughout the life cycle of malaria parasites [37]. Hence, cultivation without the addition of 2 mM CaCl_2_ for 48 h may have decreased parasite metabolism beyond protein transport alone. Therefore, the effects of low Ca^2+^ conditions on parasite metabolism must also be considered.

This study revealed that the uptake of serum proteins was detected in PV lysate treated with CytD and MycB, however, was lower or not detected, in PV lysate treated with Jas (Fig. 4c). Although Jas increases the assembly and stabilization of actin filaments [34], it is unclear whether this causes direct inhibition of protein uptake as parasite development was arrested at an early stage (Fig. 4a, b, Jas and c, SERA5). Hence, although the current study did not sufficiently demonstrate a relationship between the uptake mechanism and endocytosis, the study performed by Smythe et al. [5] reported that the uptake of preloaded recombinant HRP is inhibited by Jas, and not by CytD, suggesting that Jas does in fact inhibit endocytosis in the parasites. Further studies are required to more clearly elucidate the role of endocytosis in the protein uptake mechanism.

A comparison of the primary structures of these three internalized proteins revealed that both protein S and prothrombin have a γ-carboxyglutamic acid-rich (Gla) domain that is responsible for high-affinity binding to Ca^2+^ [38, 39]. This domain anchors proteins to cell membranes that expose phosphatidylserine (PS) in a Ca^2+^-dependent manner [40]. In eryptosis, a type of programmed cell death for RBCs, Ca^2+^ influx into iRBCs exposes PS residues on the outer membrane leaflet [41]. It is speculated that the serum proteins that bind to PS on the outer membrane leaflet of RBCs pass through the membrane via unknown mechanisms. Vitronectin does not contain this domain, but rather forms a complex with thrombin in serum [42] and may be taken up as the complex. Other Gla domain-containing proteins, such as Factor VII, IX, X, XIV, and inter-alpha-trypsin inhibitor heavy chain H2 had considerably lower uptake ratios (Table 1). These findings imply that the Gla domain and other factors determine uptake ability.

In terms of the total amount of proteins internalized in iRBCs, prothrombin and serum albumin were considerably more abundant than the other proteins (Table 1) (TSC in isolated parasite lysate: prothrombin, 135.46; serum albumin, 128.56). Although serum albumin is necessary for parasite growth [43, 44], the uptake of prothrombin is not essential in vitro (Figs. 2 and 3). Considering that the parasite-dependent activation of coagulation causes severe malarial complications [18], our findings provide further insights into the role of thrombin in eliciting symptoms during parasitic infection. In particular, the direct activation of prothrombin in isolated parasite lysate (Fig. 5c) implies that DIC was mediated in response to this reaction. In addition, it is also possible that activated thrombin detaches iRBCs from the cell surface. Activated thrombin cleaves the major parasite adhesive protein *P. falciparum* erythrocyte membrane protein 1 (PfEMP1) on the surface of iRBCs, dramatically reducing the adhesion of iRBCs and detaching iRBCs [20]. Hence, parasites may promote their cytoadherence by selectively taking up serum thrombin that cleaves PfEMP1, or by eliminating host factors that could limit cytoadherence.

Protease(s) released from the digestive vacuole activate complement factors and coagulation [45]. In the present study, although the coagulation assay demonstrated prothrombin activation in PDS-culture supernatant (Fig. 5a), it remains unclear whether the activated thrombin was released from the iRBCs or the released prothrombin was activated by the protease(s) in the PDS-culture supernatant.

Prothrombin is cleaved and activated by serine protease Factor Xa [46], implying that a parasite-derived serine protease cleaves and activates internalized prothrombin. The *P. falciparum* genome encodes 14 serine proteases, including two chymotrypsin-like, three subtilisin-like (known as PfSUB-1 to -3), and nine rhomboid protease clans [47]. Since Factor Xa belongs to the PA clan (proteases of mixed nucleophile, superfamily A) and has a chymotrypsin-like fold, two chymotrypsin-like proteases (PF3D7_0807700 and PF3D7_0812200) are candidates for prothrombin digestion. These genes are highly expressed during the late ring stage to late schizont stage (PlasmoDB: http://plasmodb.org/plasmo/). PF3D7_0807700, known as PfDegP, plays an essential role in resistance to thermo-oxidative stress, thereby affecting parasite growth and development [48]. Antibodies against DegP showed anti-plasmodial activity against erythrocytic stage parasites in vitro [48, 49], suggesting that PfDegP may be a potential target for new antimalarial therapies.

The present study showed that thrombin activated by parasite-derived proteases promotes coagulation (Fig. 5b). The previous study demonstrated that vitronectin binds directly to the P47 domain of SERA5, thereby camouflaging the parasite and enabling it to evade the host immune system [8]. Although protein S is significantly taken up, its molecular function for parasite development remains uncharacterized. Protein S is an anticoagulant and may be cleaved and inactivated in iRBCs. Further studies are required to clarify the role of protein S in *P. falciparum* infection.

## Conclusions

Serum proteins were selectively taken up by the parasites in culture conditions with sufficient Ca^2+^ levels. Among them, prothrombin was activated and caused blood coagulation. These findings suggest that the serum proteins taken up by *P. falciparum* parasites are associated with malaria pathogenicity.

## Supplementary information

**Additional file 1: Fig. S1.** Schematic illustration of the two procedures for serum preparation. **Fig. S2.** Serum proteins in RBCs infected with rodent malarial parasite in vivo. Confocal microscopy images showing the localization of vitamin K-dependent protein S (protein S), prothrombin, and vitronectin (red), and DNA (blue) in the RBCs of mice infected with the rodent malarial parasite *Plasmodium yoelii in vivo*. All samples were fixed with the methanol fixation method. Primary [anti-protein S pAb, anti-prothrombin pAb, anti-vitronectin pAb, or rabbit IgG antibody (control; Catalogue number: 30000-0-AP from Proteintech, Rosemont, IL, USA)] and secondary (anti-rabbit IgG-Alex Flour594) antibodies were suitably diluted (1:200) and used. Images were captured using a BZ-X710 fluorescence microscope (Keyence, Osaka, Japan). “Rabbit IgG” refers to the isotype control antibody. Arrows and arrowheads indicate the iRBCs and non-iRBCs, respectively. The scale bar represents 5 µm. **Fig. S3.** Serum proteins in RBCs infected with the CDC1 and W2 strains. (a) CDC1 strain. (b) W2 strain. These strains were cultured under the same conditions as those for the 3D7 strain. (Left panels) Serum proteins in the lysate of iRBCs cultured in NCS- or PDS-containing medium. Samples were collected using the precipitation method. (Right panels) Serum proteins in NCS and PDS. The asterisk indicates the putative degradation product. **Fig. S4.** Confirmation of the generation of Factor Xa for coagulation. Western blotting analysis of Factor Xa, the activated form of Factor X. No signal corresponding to Factor Xa (estimated molecular weight of approximately 28.5 kDa) was detected by anti-Factor Xa pAb (ab111171; Abcam) even when the blot was overexposed. The intensity of the image of Factor X shown in Fig. 5c (i) has been increased. The asterisk indicates the putative degradation product.

## Data Availability

The datasets generated during and/or analysed during the current study are available from the corresponding author on reasonable request.

## Abbreviations

EDTA: Ethylenediaminetetraacetic acid
EGTA: Ethylene glycol-bis(β-aminoethyl ether)-*N*,*N*,*N*ʹ,*N*ʹ-tetraacetic acid
sodium citrate: Tri-sodium citrate dehydrate
Jas: Jasplakinolide
CytD: Cytochalasin D
MycB: Mycalolide B
RBC: Red blood cell
LC–MS/MS: Liquid chromatography–mass spectrometry/mass spectrometry
PDS: Plasma-derived serum
NCS: Naturally coagulated serum
PV: Parasitophorous vacuole
RIMD: Research Institute for Microbial Diseases
Protein S: Vitamin K-dependent protein S
AHSG: Alpha-2-HS-glycoprotein
C4BPA: C4b-binding protein alpha chain
SERA5: Serine repeat antigen 5
PMSF: Phenylmethylsulfonyl fluoride
DE: Dabigatran etexilate
PBS: Phosphate-buffered saline
