# Supplementary material for: The malaria parasite Plasmodium falciparum in red blood cells selectively takes up serum proteins that affect host pathogenicity
Source: Malar J. 2020 Apr 15;19:155. doi: 10.1186/s12936-020-03229-1 (PMC7161009; doi:10.1186/s12936-020-03229-1)
Supplement: Supplementary file 1 — Additional file 1: Fig. S1. Schematic illustration of the two procedures for serum preparation. Fig. S2. Serum proteins in RBCs infected with rodent malarial parasite in vivo. Confocal microscopy images showing the localization of vitamin K-dependent protein S (protein S), prothrombin, and vitronectin (red), and DNA (blue) in the RBCs of mice infected with the rodent malarial parasite Plasmodium yoelii in vivo. All samples were fixed with the methanol fixation method. Primary [anti-protein S pAb, anti-prothrombin pAb, anti-vitronectin pAb, or rabbit IgG antibody (control; Catalogue number: 30000-0-AP from Proteintech, Rosemont, IL, USA)] and secondary (anti-rabbit IgG-Alex Flour594) antibodies were suitably diluted (1:200) and used. Images were captured using a BZ-X710 fluorescence microscope (Keyence, Osaka, Japan). “Rabbit IgG” refers to the isotype control antibody. Arrows and arrowheads indicate the iRBCs and non-iRBCs, respectively. The scale bar represents 5 µm. Fig. S3. Serum proteins in RBCs infected with the CDC1 and W2 strains. (a) CDC1 strain. (b) W2 strain. These strains were cultured under the same conditions as those for the 3D7 strain. (Left panels) Serum proteins in the lysate of iRBCs cultured in NCS- or PDS-containing medium. Samples were collected using the precipitation method. (Right panels) Serum proteins in NCS and PDS. The asterisk indicates the putative degradation product. Fig. S4. Confirmation of the generation of Factor Xa for coagulation. Western blotting analysis of Factor Xa, the activated form of Factor X. No signal corresponding to Factor Xa (estimated molecular weight of approximately 28.5 kDa) was detected by anti-Factor Xa pAb (ab111171; Abcam) even when the blot was overexposed. The intensity of the image of Factor X shown in Fig. 5c (i) has been increased. The asterisk indicates the putative degradation product. [file 12936_2020_3229_MOESM1_ESM.docx]

**Fig. S1**

**
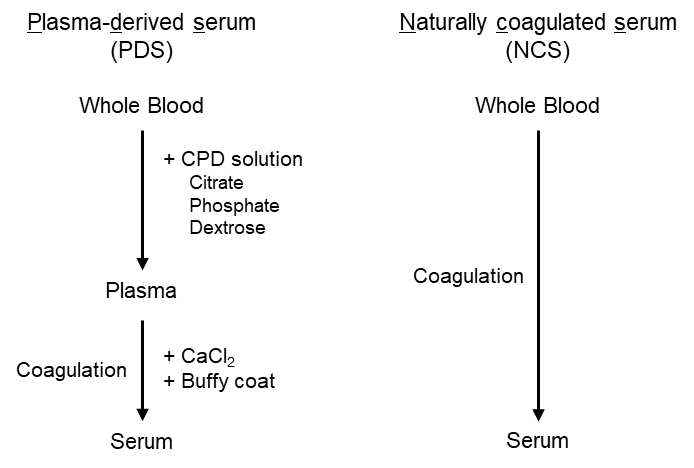
**

**Fig. S1. Schematic illustration of the two procedures for serum preparation.**

**
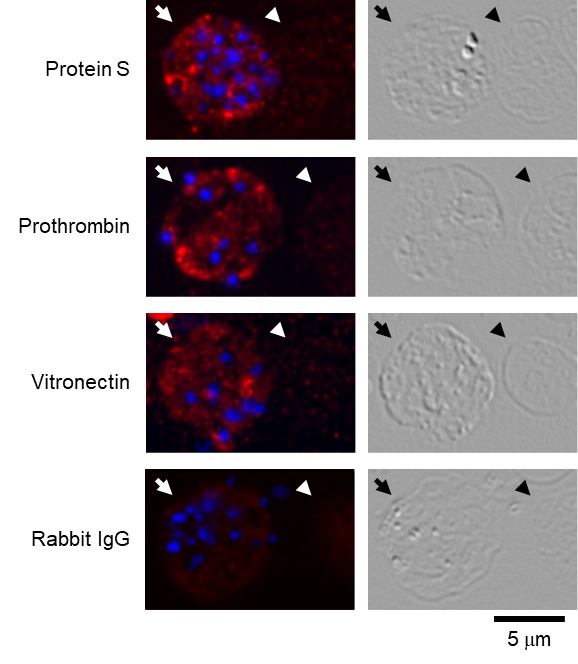
Fig. S2**

**Fig. S2. Serum proteins in RBCs infected with rodent malarial parasite *in vivo*.** Confocal microscopy images showing the localisation of vitamin K-dependent protein S (protein S), prothrombin, and vitronectin (red), and DNA (blue) in the RBCs of mice infected with the rodent malarial parasite *Plasmodium yoelii in vivo*. All samples were fixed with the methanol fixation method. Primary [anti-protein S pAb, anti-prothrombin pAb, anti-vitronectin pAb, or rabbit IgG antibody (control; Catalogue number: 30000-0-AP from Proteintech, Rosemont, IL, USA)] and secondary (anti-rabbit IgG-Alex Flour594) antibodies were suitably diluted (1:200) and used. Images were captured using a BZ-X710 fluorescence microscope (Keyence, Osaka, Japan). “Rabbit IgG” refers to the isotype control antibody. Arrows and arrowheads indicate the iRBCs and non-iRBCs, respectively. The scale bar represents 5 μm.

**
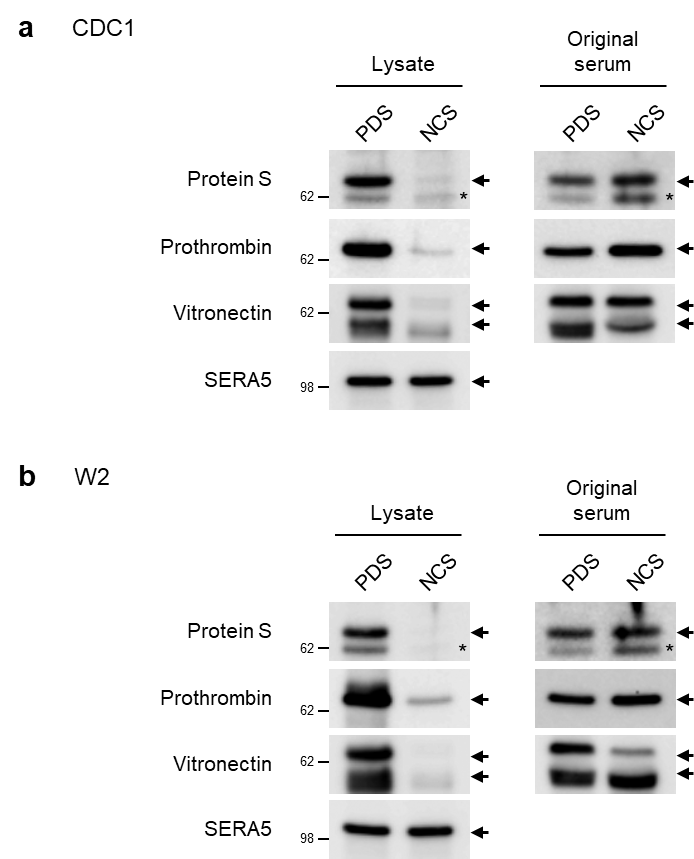
Fig. S3**

**Fig. S3. Serum proteins in RBCs infected with the CDC1 and W2 strains.** (a) CDC1 strain. (b) W2 strain. These strains were cultured under the same conditions as those for the 3D7 strain. (Left panels) Serum proteins in the lysate of iRBCs cultured in NCS- or PDS-containing medium. Samples were collected using the precipitation method. (Right panels) Serum proteins in NCS and PDS. The asterisk indicates the putative degradation product.

**
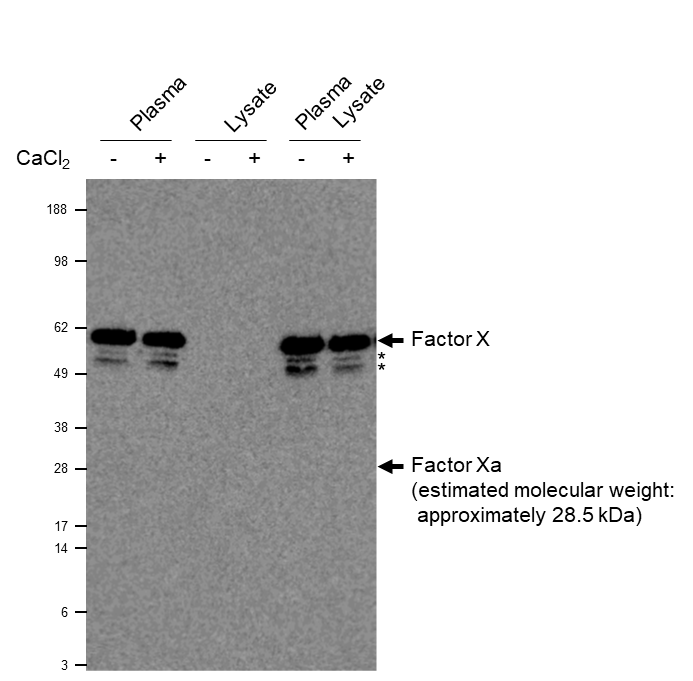
Fig. S4**

**Fig. S4. Confirmation of the generation of Factor Xa for coagulation.** Western blotting analysis of Factor Xa, the activated form of Factor X. No signal corresponding to Factor Xa (estimated molecular weight of approximately 28.5 kDa) was detected by anti-Factor Xa pAb (ab111171; Abcam) even when the blot was overexposed. The intensity of the image of Factor X shown in Fig. 5c (i) has been increased. The asterisk indicates the putative degradation product.
